# Osteoclasts Control Osteoblast Chemotaxis via PDGF-BB/PDGF Receptor Beta Signaling

**DOI:** 10.1371/journal.pone.0003537

**Published:** 2008-10-27

**Authors:** Maria Arantzazu Sanchez-Fernandez, Anne Gallois, Thilo Riedl, Pierre Jurdic, Bernard Hoflack

**Affiliations:** 1 Biotechnological Center, Dresden University of Technology, Dresden, Germany; 2 Institut de Génomique Fonctionnelle de Lyon, Ecole Normale Supérieure de Lyon, CNRS, INRA, Université Claude Bernard Lyon 1, Lyon, France; Max Planck Institute of Molecular Cell Biology and Genetics, Germany

## Abstract

**Background:**

Bone remodeling relies on the tightly regulated interplay between bone forming osteoblasts and bone digesting osteoclasts. Several studies have now described the molecular mechanisms by which osteoblasts control osteoclastogenesis and bone degradation. It is currently unclear whether osteoclasts can influence bone rebuilding.

**Methodology/Principal Findings:**

Using *in vitro* cell systems, we show here that mature osteoclasts, but not their precursors, secrete chemotactic factors recognized by both mature osteoblasts and their precursors. Several growth factors whose expression is upregulated during osteoclastogenesis were identified by DNA microarrays as candidates mediating osteoblast chemotaxis. Our subsequent functional analyses demonstrate that mature osteoclasts, whose platelet-derived growth factor bb (PDGF-bb) expression is reduced by siRNAs, exhibit a reduced capability of attracting osteoblasts. Conversely, osteoblasts whose platelet-derived growth factor receptor β (PDGFR-β) expression is reduced by siRNAs exhibit a lower capability of responding to chemotactic factors secreted by osteoclasts.

**Conclusions/Significance:**

We conclude that, *in vitro* mature osteoclasts control osteoblast chemotaxis via PDGF-bb/PDGFR-β signaling. This may provide one key mechanism by which osteoclasts control bone formation *in vivo*.

## Introduction

Bones undergo remodeling to maintain the mass, the shape and the physical properties of the skeleton. Two major cell types, the bone-forming osteoblasts and the bone-resorbing osteoclasts contribute to this process, which occurs continuously throughout life. The balance between bone formation and degradation is normally tightly controlled but it becomes deregulated, shifting towards more degradation under pathological conditions or during aging, thereby leading to osteoporosis [1], [2]. This tight balance implies the existence of mechanisms coordinating the differentiation of osteoblasts and osteoclasts as well as their migration to locations where they function.

The mechanisms by which osteoblasts control the differentiation of hematopoietic osteoclast precursors towards mature multinucleated cells, i.e. osteoclastogenesis, are now established [3]. Osteoblasts and stromal cells control bone degradation by expressing the Macrophage-Colony Stimulating Factor (M-CSF) required for the proliferation of osteoclast precursors and the Receptor for Activation of NF-kB Ligand (RANKL), a TNF family member triggering their differentiation [1], [3]–[7]. The mechanisms controlling the differentiation of mesenchymal stromal cells (MSCs) towards osteoblasts, i.e osteoblastogenesis, are less understood. During the past years, several signaling molecules have been implicated in bone development and osteogenesis [8]. These include fibroblast growth factors, parathyroid hormone complexes [9], bone morphogenetic proteins (BMPs) [10], Canonical Wnt [11]–[14], Indian hedgehog [15] and epidermal growth factor [16]. However, the cell types producing these factors have remained elusive. There is evidence however, that osteoclasts can control osteoblast function. Ephrin B2 expressed by osteoclasts and its Ephrin B4 receptor expressed by osteoblasts allow bidirectional signaling [17]. Signaling through Ephrin B4 into osteoblasts enhances osteogenic differentiation whereas signaling through Ephrin B2 into osteoclast precursors suppresses osteoclast differentiation. It has also been reported that the v-ATPase V_0_ subunit D2 is not only involved in osteoclast fusion but also regulates the secretion by osteoclasts of still unidentified factors that inhibit the differentiation of osteoblast precursors into mature cells [18].

During development, osteoblasts must colonize the cartilage that will be replaced by bone. During adulthood, bone remodeling and repair require the migration of osteoblasts to bone areas that need to be rebuilt. This latter process also requires the mobilization of their progenitors residing together with MSCs into niches of the bone marrow [19], [20]. A plethora of growth factors used as recombinant proteins, in particular BMPs, platelet-derived growth factor (PDGF), vascular endothelial growth factor (VEGF) and leukemia inhibitory factor (LIF), have been shown to exhibit *in vitro* chemotactic activities towards various cell types including osteoblasts and their progenitors [21], [22]. However, it is still unclear which cell types in the bone matrix secrete the factors able to attract bone rebuilding cells, which growth factors can function in this context and how the migration of bone rebuilding cells is coordinated with bone digestion.

We tested here the hypothesis that osteoclasts can control osteoblast chemotaxis. Using *in vitro* cell systems of osteoclastogenesis and osteoblasto-genesis, we show that mature osteoclasts secrete PDGF-bb, which is recognized by PDGF receptor β (PDGFR-β) on the surface of osteoblasts and regulates osteoblast chemotaxis. Thus, this *in vitro* study provides the first molecular basis for further investigating the importance of the PDGF-bb/ PDGFR-β signaling pathway in bone remodeling *in vivo*.

## Results

In order to test whether osteoclasts can signal to osteoblasts, we used *in vitro* cell systems of osteoclastogenesis or osteoblastogenesis. First, we used the mouse monocyte/macrophage-like Raw264.7 cells, which differentiate *in vitro* towards mature osteoclasts upon stimulation with the osteoclastogenic cytokine RANKL. Raw264.7 cell-derived osteoclasts conform to conventional assays for osteoclast function as primary osteoclasts isolated from bones. Primary osteoclast precursors from mouse bone marrow and derived osteoclasts were also used. Second, we used the mouse pre-osteoblastic MC3T3-E1 cells that differentiate *in vitro* towards mature osteoblasts upon stimulation with chemical cocktails containing dexamethasone, ascorbic acid and β-glycerophosphate. The MC3T3-E1-derived osteoblasts express the typical markers of osteoblasts isolated from bones.

### Osteoclasts secrete factors attracting osteoblasts

**Figure 1:**
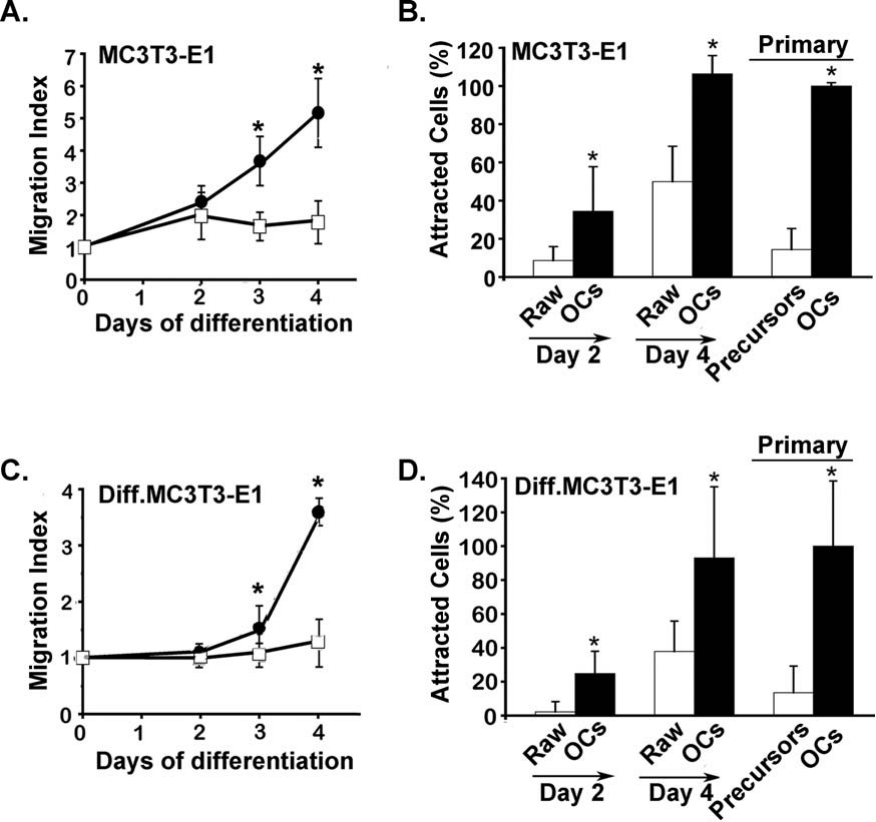
Chemotaxis response of osteoblasts and precursors to factors secreted by osteoclasts. Raw264.7 cells were grown in the presence or absence of RANKL. Conditioned media were collected every day. The chemotactic activity of the corresponding conditioned media towards pre-osteoblastic MC3T3-E1 cells and derived osteoblasts was measured as described under materials and methods. Migration Index of mouse pre-osteoblastic MC3T3-E1 cells (A) and derived osteoblasts (diff MC3T3-E1) (C) in response to conditioned media of Raw264,7 cells (□) and derived osteoclasts (•). Chemotaxis of pre-osteoblastic MC3T3-E1 cells (B) and derived osteoblasts (D) by conditioned media of primary osteoclasts and their precursors. For comparison, the chemotactic activity of conditioned media from Raw264, 7 cells and derived osteoclasts collected after 2 and 4 days of differentiation are shown. Shown are mean values±S.D. of four independent experiments performed in triplicates. *P* values from ANOVA tests equal or less than 0.05 were considered significant and are marked with an asterisk (*).

We first tested whether the conditioned medium of Raw264.7 cells or Raw264.7-derived osteoclasts could attract pre-osteoblastic MC3T3-E1 cells or MC3T3-E1-cell derived osteoblasts. For this, we used the Boyden chamber assay to measure chemotaxis. Figure 1A shows that the chemotactic activity of Raw264.7 cells towards MC3T3-E1 cells was rather low. However, their chemotactic activity increased with time when they were differentiated towards osteoclasts upon stimulation with RANKL. After 4 days of RANKL-induced differentiation, the conditioned medium of Raw-derived osteoclasts exhibited a clear chemotactic activity towards MC3T3-E1 cells (Fig. 1A). This activity was also observed with the conditioned medium of osteoclasts derived from bone marrow progenitors stimulated by M-CSF and RANKL (Fig. 1B). The conditioned medium of Raw264.7-derived osteoclasts also exhibited a chemotactic activity towards MC3T3-E1-derived osteoblasts, although to a lower extent (Fig. 1C). The chemotatic index of mature osteoclast conditioned media was two fold higher towards pre-osteoblastic MC3T3-E1 cells when compared to MC3T3-E1 cell-derived osteoblasts. Similar results were obtained with the conditioned media of primary osteoclasts derived from bone marrow progenitors (Fig. 1D). We conclude from these results that, during their differentiation osteoclasts derived from Raw264.7 cells or primary bone marrow progenitors acquire the capability of secreting chemotactic factors able to attract osteoblast precursors and, to a lower extent mature osteoblasts.

### Identification of putative chemotactic factors

**Figure 2:**
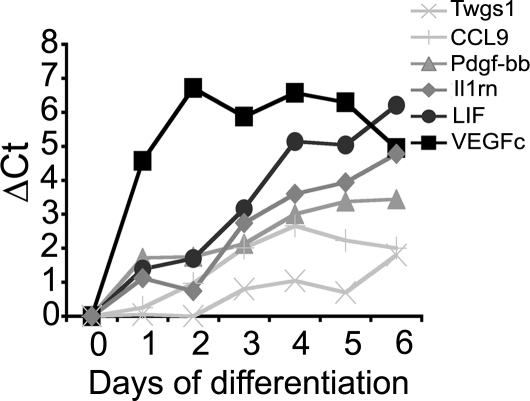
Gene expression levels during osteoclastogenesis. Shown are the expression levels of (▪) VEGFc, (•) LIF, (♦) IL1ra, (▴) PDGF-bb, (+) CCL9 and (×) Twgs1 during the RANKL-induced differentiation of Raw264,7 cells. Quantitative RT-PCR analyses were performed as described under materials and methods. Data are expressed in variations of ΔCt.

The results described above strongly suggest that the genes encoding chemotactic factors secreted by mature osteoclasts are upregulated during osteoclastogenesis. To identify candidates, we took advantage of our previous DNA microarray analyses comparing the gene expression patterns of Raw264.7 cells and derived osteoclasts [23]. We identified eighteen genes upregulated during osteoclastogenesis, encoding secreted proteins, in particular PDGF-bb, vascular endothelial growth factor c (VEGFc), leukemia inhibitory factor (LIF) and cytokines such as the chemokine (C-C motif) ligand 9 (CCL9), interleukin-1 receptor antagonist (IL-1ra) and Twisted gastrulation protein 1 (Twgs1). Quantitative RT-PCR performed on Raw264. 7 cells and derived osteoclasts shows that, indeed the expression of PDGF-bb, VEGFc, LIF and IL-1ra was substantially upregulated during osteoclastogenesis (a 13, 36, 73 and 25 fold increase, respectively) whereas that of CCL9 and Twgs 1 was upregulated only 2–4 fold (Table 1). Similar results were obtained for osteoclasts derived from primary osteoclast progenitors from bone marrow, in which PDGF-bb and LIF expression were even more strongly upregulated (a 190 and 90 fold increase respectively) (Table 1). Figure 2 shows that the expression of PDGF-bb, LIF and IL-1ra increased regularly during the RANKL-induced differentiation of Raw264.7 cells whereas that of VEGFc was maximal after two days of differentiation and remained constant.

**Table 1:** Growth factors levels during osteoclastogenesis.

Gene	Changes in mRNA amounts (folds)	
	Raw264.7 derived osteoclast	Primary osteoclasts
PDGF-bb	13	194
VEGFc	36	6.5
LIF	73	90
IL1ra	25	6
CCL9	4	15
Twgs protein 1	2	2

Raw 264.7 cells and primary osteoclast precursors from bone marrow were differentiated towards osteoclasts with RANKL as indicated under materials and methods. After 6 days, changes in gene expression were detected by quantitative RT-PCR as indicated under materials and methods and normalized according to GAPDH expression.

### PDGF-bb secreted by osteoclasts mediates osteoblast chemotaxis

**Figure 3:**
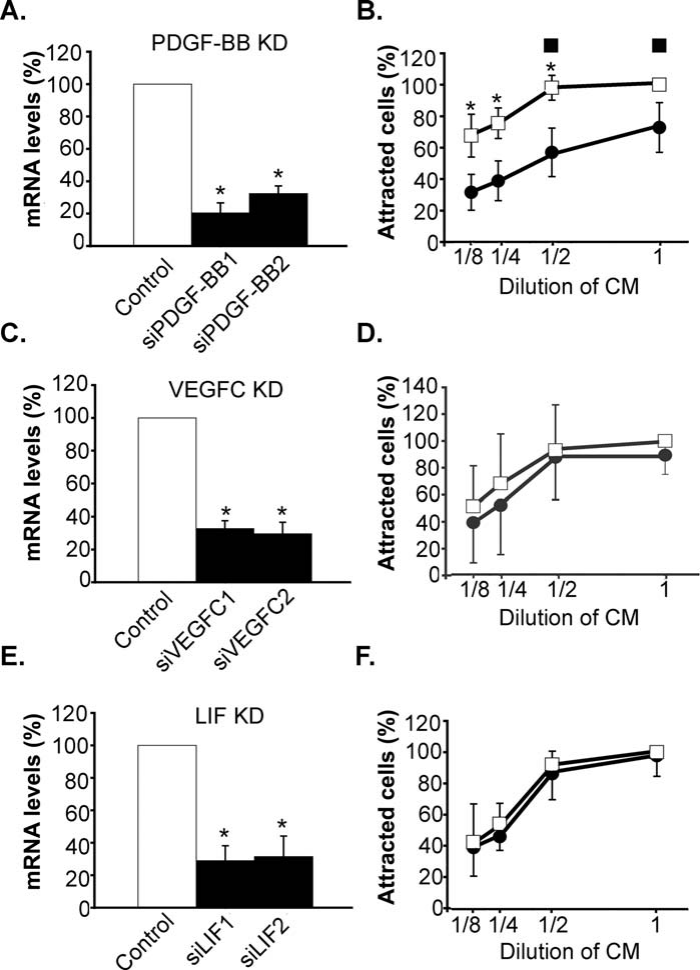
PDGF-bb secreted by osteoclasts triggers osteoblast chemotaxis. Genes encoding PDGF-bb, VEGFc, LIF were silenced in Raw264,7 cell-derived osteoclasts as described under materials and methods. Conditioned media of treated osteoclasts were collected and different dilutions were tested for their chemotactic activity towards pre-osteoblastic MC3T3 cells. Knockdown efficiencies of PDGF-bb (A), VEGFc (C) and LIF (E) in Raw264,7 cell-derived osteoclasts were determined by quantitative RT-PCR. The knockdown efficiencies were 74%, 71% and 70% respectively (p<0,00001, ANOVA). Chemoattraction of pre-osteoblastic MC3T3 cells by different dilutions of conditioned media of Raw264,7 cells (•) or Raw264,7 cell-derived osteoclasts (□) in which the expression of PDGF-bb (B), VEGFc (D) and LIF (F) were silenced (p<0,0001, ANOVA). (▪ in B) Rescue of PDGF-bb knockdown in osteoclasts: conditioned media of siRNA-treated osteoclasts were supplemented with 10 ng/ml recombinant human PDGF-bb. Data points represent the average of 5 experiments.

To identify the chemotatic factor(s) secreted by mature osteoclasts, we used a siRNA-based strategy. Raw264.7-derived osteoclasts were first electroporated in the presence of siRNA probes and then maintained in culture. After two days, the silencing efficiencies were determined by quantitative RT-PCR and the conditioned media of siRNA-treated osteoclasts were tested for their chemotactic activity. As shown in figure 3, an efficient siRNA-mediated depletion of PDGF-bb, VEGFc or LIF (≈80%) could be achieved in Raw-derived osteoclasts. A reduction in PDGF-bb expression in Raw-derived osteoclasts resulted in a ≈50% reduction in the ability of their conditioned medium to attract pre-osteoblastic MC3T3-E1 cells at every concentration tested. The residual chemotactic activity of pre-osteoblastic MC3T3-E1 cells by conditioned media of siRNA-treated osteoclasts most likely reflects the presence of low amounts of PDGF-bb still secreted by these cells. In addition, this loss of chemotactic activity after PDGF-bb knockdown could be rescued by the addition of recombinant PDGF-bb, reaching the same chemotactic activity of conditioned media of non-siRNA-treated osteoclasts (Fig. 3B). In contrast, a 80% reduction in VEGFc or LIF expression remained without any effect on chemotaxis of MC3T3-E1 cells (Fig. 3C–F). Because recombinant CCL9, IL1ra and Twgs1 did not modify the chemotactic activity of MC3T3-E1 cells (data not shown), they were not further considered. We conclude from these results that PDGF-bb secreted by Raw264.7-derived osteoclasts acts as a potent chemotatic agent towards preosteoblastic MC3T3-E1 cells.

### PDGFR-β on the surface of osteoblasts binds PDGF-bb secreted by osteoclasts

PDGF-bb binds either to PDGFR-β or PDGFR-α homodimers or to PDGFR-α/β heterodimers [24]. PDGFR-α and PDGFR-β mRNAs could be detected in pre-osteoblastic MC3T3-E1 cells, suggesting that these receptors are expressed on their cell surface. Using quantitative RT-PCR, we determined their expression levels during osteoblastogenesis. Table 2 shows that the expression level of both PDGFR-α and PDGFR-β decreased substantially when pre-osteoblastic MC3T3-E1 cells were differentiated towards osteoblasts upon induction with chemical cocktails (a ≈80% reduction within 8 days of differentiation). For comparison, we also followed variations in the expression of VEGFR-3, the main VEGF receptor expressed in pre-osteoblastic MC3T3-E1 cells. Its expression also decreased during MC3T3-E1 cell differentiation (Table 2) as that of the LIF receptor (not shown).

**Table 2:** Growth factor receptor expression levels during osteoblastogenesis.

Differentiation	mRNA expression levels (%)		
	PDGFR-β	PDGFR-α	VEGFR-3
Day 0	100	100	100
Day 2	35,7	50	50
Day 4	26.3	27.8	50
Day 6	11.2	20	47.6
Day 8	11.7	16.1	33.4

Differentiation of MC3T3-E1 cells to osteoblasts was induced with dexamethasone, ascorbic acid and β-glycerophosphate during the indicated period of time. Changes in gene expression levels were detected by quantitative RT-PCR as indicated under materials and methods and normalized according to GAPDH expression.

**Figure 4:**
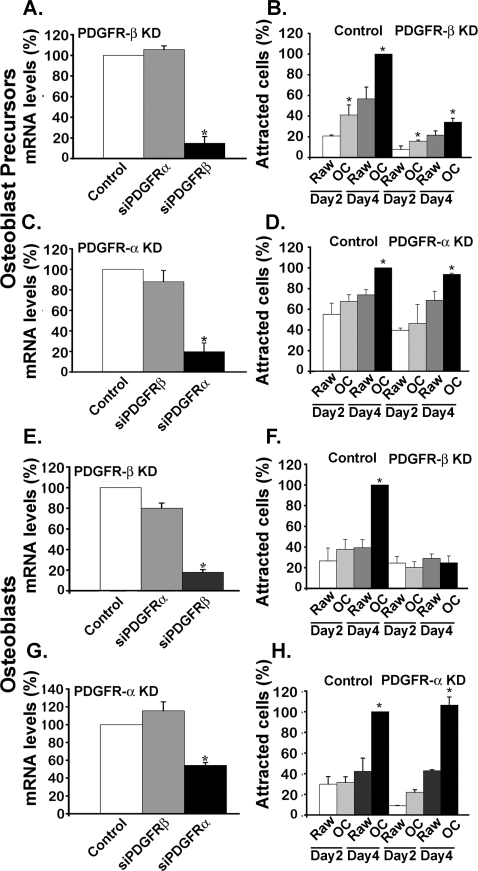
PDGFR-β of pre-osteoblastic MC3T3-E1 cells and derived osteoblasts relays osteoclast signaling. PDGFR-β and PDGFR-α genes were silenced in MC3T3-E1 cells (A, B) and derived osteoblasts (F, H) as described under materials and methods. The knockdown efficiencies were 85%, 80% (p<0.0001), 82% and 50% (p<0.000001 and p = 0.01), respectively. (B, D, F, H) The siRNA-treated cells were tested for their chemotactic activities towards conditioned media of Raw264, 7 cells and derived osteoclasts after 2 or 4 days of RANLK induction (p<0.001 and p<0.0001 respectively). Shown are mean values±S.D. of three independent experiments performed in triplicates.

To identify the PDGFR that binds PDGF-bb secreted by Raw264.7-derived osteoclasts, we also followed a siRNA-based strategy. Pre-osteoblastic MC3T3-E1 cells or derived osteoblasts were treated with siRNAs. After 2–3 days, the knockdown efficiencies were determined by quantitative RT-PCR and the treated cells were tested for their ability to be attracted by the conditioned media of Raw264.7 cells or derived osteoclasts. Figure 4 shows that the knockdown of PDGFR-β in pre-osteoblastic MC3T3-E1 cells, leading to a ≈90% reduction in its expression without affecting PDGFR-α expression, resulted in a loss of their attraction by conditioned media from Raw264.7-derived osteoclasts. In contrast, the knockdown of PDGFR-α in pre-osteoblastic MC3T3-E1 cells, leading to a ≈90% reduction in its expression without affecting PDGFR-β expression, did not affect their attraction by conditioned media from Raw264.7-derived osteoclasts (Fig. 4C & 4D). Similar results were obtained when PDGFR-α or β expression were reduced in MC3T3-E1-derived osteoblasts (Fig. 4 E–H). Furthermore, pre-osteoblastic MC3T3-E1 cells and derived osteoblasts with reduced PDGFR-β expression were unable to respond to recombinant PDGF-bb, thereby providing additional support to the specificity of these interactions (Figure S1). We conclude from these results that PDGFR-β is the receptor isoform, which binds PDGF-bb at the cell surface of pre-osteoblastic MC3T3-E1 cells and derived osteoblasts.

## Discussion

Our study shows that osteoclast precursors up-regulate the expression of several growth factors during osteoclastogenesis and that PDGF-bb is the only secreted growth factor triggering *in vitro* the chemotaxis of pre-osteoblastic cells and, to a lower extent that of the corresponding derived osteoblasts. This lower reactivity of osteoblasts is likely due to a lower expression of PDGFR-β, the unique PDGFR isoform that binds PDGF-bb and triggers their chemotaxis. Thus, PDGF-bb/PDGFR-β signaling controls the chemo-attraction of osteoblasts by osteoclasts *in vitro*.

Chemotaxis and cell migration are key biological events necessary for tissue formation and remodeling. Thus, a continuous supply of osteoblastic cells is required to build the bone tissue at specific sites during development and for bone remodeling and fracture healing during adulthood. Our study demonstrates that mature osteoclasts derived from Raw264.7 cells and primary progenitors isolated from the bone marrow secrete factors able to induce the chemotactic response of both preosteoblastic MC3T3-E1 cells and derived osteoblasts *in vitro*. This chemotactic activity is clearly dependent upon the differentiation state of osteoclasts since osteoclast precursors fail to produce strong chemotactic responses. Therefore, the genes encoding these chemokines must be strongly upregulated during the RANKL-dependent differentiation of osteoclast precursors. DNA microarrays comparing gene expression between mature osteoclasts and their precursors identified a group of several genes encoding secreted proteins that are upregulated during osoteoclastogenesis. Similar results were obtained using two different systems of osteoclastogenesis, either mouse Raw264.7 cells [23 and this study] or primary osteoclast progenitors from mouse bone marrow, and were confirmed by quantitative RT-PCR analyses. Similar results were also obtained with human systems of osteoclastogenesis (A. Gallois and P. Jurdic, unpublished observations). Some of these genes encode known growth factors, in particular PDGF-bb, VEGFc and LIF. A role for PDGF-bb in bone formation has been postulated since recombinant PDGF-bb, as other PDGF isoforms or BMP-2 and BMP-4 isoforms, has been shown to be a powerful chemoattractant for mesenchymal stromal cells and osteoblasts *in vitro* [21]. It has been proposed that LIF could modulate the chemotactic activity of PDGF [25]. However, we found that the pre-treatment of osteoblasts or osteoclasts with LIF did not modify the chemotactic response of osteoblasts towards PDGF-bb *in vitro* (unpublished observation). Other studies have indicated a critical role for VEGF during the process of endochondral ossification, in particular in coupling cartilage resorption with bone formation [26]. VEGFa has been shown to exhibit *in vitro* chemotactic activities towards primary osteoblasts [22]. Thus, our results confirm previous findings showing that recombinant PDGF-bb can attract osteoblasts *in vitro* and also extend these findings by identifying the mature osteoclasts as a source of this growth factor. In contrast, it appears that VEGFc secreted by osteoclasts does not exhibit chemotactic activity towards osteoblasts or their precursors. VEGFc secreted by osteoclasts was recently shown to function as an autocrine factor regulating osteoclast activity [27]. It remains possible that the other secreted cytokines, as well as VEGFc, influence the behavior of the other cell types involved in bone metabolism, a hypothesis, which remains to be tested.

PDGF-bb, as well as other PDGF isoforms, binds with similar affinities to PDGF receptors consisting of either α, β homodimers or α/β heterodimers [24]. It has been unclear, which PDGFR isoform triggers the signaling cascade leading to the chemotaxis of osteoblasts or their precursors. This issue has been addressed using anti-PDGFR-α or PDGFR-β blocking antibodies whose specificity has remained controversial [21]. The inactivation of PDGF-bb or PDGFR-β genes results in severe cardiovascular, renal, placental, and hematologic disorders and is therefore embryonic lethal, rendering phenotypic analyses difficult [28], [29], [24]. Our study clearly identifies PDGFR-β at the surface of pre-osteoblastic MC3T3-E1 cells and derived osteoblasts as the receptor binding osteoclastic PDGF-bb, thereby triggering their chemotaxis *in vitro*. The knockdown of PDGFR-β in osteoblasts or that of PDGF-bb in mature osteoclasts led to the same phenotype i.e. a loss of migration of osteoblasts. In contrast, the knockdown of PDGFR-α remained without any effect on migration. Thus, our *in vitro* study strongly suggests that PDGF-bb/PDGFR-β signaling is critical for bone remodeling and/or fracture healing *in vivo* [30]. Therefore, our study could provide a molecular basis for investigating the importance of this signaling pathway in the context of bone remodeling. It is currently unknown how PDGFR-β signaling can trigger the re-organization of actin dynamics required for cell movement of osteoblasts and their precursors. In mesenchymal stromal cells, Rho GTPases have been implicated in this process [30]. In other cell types, the PI-3 kinase and the Rac GTPase have been implicated in actin remodeling triggered by PDGFR-β signaling [31], a process in which the Garb1scaffolding/docking protein and Grb2 have also been involved [32] as well as Nck and p130Cas [33]. Whereas this aspect needs to be addressed in more details, one can anticipate that some components of this signaling cascade are downregulated or differently regulated during osteoblastogenesesis.

An interesting paradigm arising from our *in vitro* study is that the production of PDGF-bb increases during osteoclastogenesis while the expression of PDGFR-β decreases during osteoblastogenesis. Mesenchymal stromal cells and osteoblast progenitors are maintained into niches of the bone marrow [20], where they also contribute to maintain hematopoietic stem cells [34]. Bone remodeling requires the mobilization and the migration of osteoblast progenitors to locations where bone needs to be rebuilt. Beside its chemotactic activity, PDGF-bb is also believed to act *in vitro* as an inhibitory factor of osteoblastogenesis [35]. Our own unpublished observations would support this contention. Thus, it could be expected that inhibition of PDGFR-β signaling in osteoblast progenitors would promote their differentiation towards osteoblasts. This latter issue has been difficult to address and has remained controversial. Imatinib mesylate, a potent inhibitor of PDGFR-β, has been found either to promote osteoblast differentiation [36] or to suppress proliferation and alter differentiation of human mesenchymal stromal cells, the osteoblast precursors [37]. As mentioned above, the knockout of PDGFR-β or PDGF-bb is lethal in mice. Therefore, more elaborated strategies are needed to explore the functional importance of PDGFR- β signaling in various tissues [28], [29], [24]. Very recently, mutant mice in which PDGFR-β was depleted with the use of an inducible Cre-loxP system were produced. The analysis of the corresponding MSCs indicated that the depletion of PDGFR-β enhances their osteogenic differentiation [38]. Our study shows that preosteoblastic cells migrate more efficiently than mature osteoblasts when stimulated by PDGF-bb, a phenomenon more than likely explained by a down regulation of PDGFR-β during osteoblastogenesis. Therefore, PDGF-bb/PDGFR-β signaling, stimulating the migration of osteoblast progenitors and concomitantly inhibiting their differentiation, could provide an efficient mechanism for a rapid mobilization of osteoblast precursors at specific sites for efficient bone rebuilding and repair. To address this point accurately, the conditional inactivation of PDGF-bb in mouse osteoclasts and that of PDGFR-β in mouse osteoblast precursors are certainly the next necessary steps to illustrate the functional importance of PDGF-bb/PDGFR-β signaling in bone remodeling and more generally the role of mature osteoclasts in controlling bone rebuilding *in vivo*.

## Materials and Methods

### Cell culture and recombinant proteins

All cell lines were from ATCC (Rockville, MD, USA). Mouse pre-osteoblastic MC3T3-E1 cells were maintained in α-MEM supplemented with 10% heat inactivated Fetal Calf Serum (FCS). Mouse myeloid Raw264.7 cells were cultured in high glucose DMEM supplemented with 10% heat inactivated FCS. Primary osteoclast precursors were obtained from bone marrow of long bones of 8 week-old C57BL/6J mice. After purification on density gradients (Eurobio), they were cultured in α-MEM supplemented with 10% heat inactivated FCS. Soluble recombinant RANKL was from Abcys (Paris, France) or produced in *Pichia* yeast as described previously [39]. Recombinant human PDGF-bb was from PeproTech EC (London, UK), human recombinant M-CSF from Prospec Tany TechnoGene (Rehovot, Israel).

### Differentiation of osteoblast and osteoclast precursors

MC3T3-E1 cells were differentiated into osteoblasts with 10^−7^ M dexamethasone, 50 µg/ml ascorbic acid and 10 mM β-glycerophosphate in α-MEM for ≈15 days. Raw264, 7 cells were differentiated into multinucleated osteoclasts in the presence of RANKL for 4–6 days as described [39]. Primary osteoclast precursors isolated from bone marrow were differentiated in the presence of M-CSF and RANKL [40]. The conditioned media of osteoclasts were collected every 24 h, centrifuged, buffered with 20 mM HEPES pH 7, 2 and kept at −80°C until further use.

### Chemotaxis Assay

Chemotactic responses were measured in triplicates using a 48-well Boyden microchemotactic chamber [41]. The lower wells of the apparatus were filled with growth factors in α-MEM containing 20 mM HEPES pH 7,2 or conditioned medium from Raw 264,7 cells or derived-osteoclasts and overlaid with a polycarbonate membrane of 5 µm pores (NeuroProbe Inc. Gaithersburg MD, USA). Cells (0.35×10^5^ MC3T3-E1, 0.45×10^5^ differentiated osteoblasts or 0.25×10^5^ 7F2 cells) in 50 µl of α-MEM were added to the upper wells. After a 3,5 h incubation at 37°C, the membrane was removed. The cells on the upper surface were discarded by gentle scraping and cells that had migrated to the other side of the membrane were fixed with 3% Paraformaldehyde, stained with toluidine blue (Sigma, Germany) and counted. The chemotactic index (CI) represents the ratio between the average number of cells migrating under given conditions and the average number of cells migrating under control conditions.

### Gene silencing in osteoclasts and osteoblasts

Raw 264,7 cells were differentiated into osteoclasts in the presence of RANKL. After 2–3 days, they were detached by incubation in PBS containing 0,5 mM EDTA. Pre-designed stealth RNAi or scrambled stealth RNAi duplexes were electroporated into osteoclasts. Electroporated cells were resuspended in medium supplemented with RANKL and maintained in culture for 48 h. Conditioned media were collected and osteoclasts were processed for total RNA isolation and protein determination. Mouse preosteoblastic MC3T3-E1 cells or chemically differentiated osteoblasts were transfected with Stealth siRNA duplex oligonucleotides using Interferin as transfection reagent. After 48 h, the cells were harvested. The total RNAs were isolated. Detailed information regarding stealth RNAi duplexes and transfection methods is given in Materials and Methods S1

### Quantitative RT-PCR Analyses

Total RNA was isolated. DNase I-treated RNAs were reverse transcribed. Quantitative RT-PCR was performed with a Stratagene Mx4000 QPCR system and the Brilliant SYBR Green QPCR kit according to the manufacturer's instructions (Stratagene, La Jolla, CA). Quantitative RT-PCR analyses were performed in triplicates, and Ct values were normalized using GAPDH. Primer sequences and detailed methods are given in Materials and Methods S1.

## Supporting Information

Materials and Methods S1 (0.05 MB DOC)

Figure S1 (8.69 MB DOC)
