# Supplementary material for: Osteoclasts Control Osteoblast Chemotaxis via PDGF-BB/PDGF Receptor Beta Signaling
Source: PLoS One. 2008 Oct 27;3(10):e3537. doi: 10.1371/journal.pone.0003537 (PMC2569415; doi:10.1371/journal.pone.0003537)
Supplement: Materials and Methods S1 — (0.05 MB DOC) [file pone.0003537.s001.doc]

### **Materials and Methods S1**

**RNAi in osteoclasts**.

For silencing of murine PDGF-bb, VEGFc and LIF, the following oligonucleotides were used.

PDGF-bb.1: 5’-GGG AUC CCA UUC CUG AGG AAC UGU A-3’,

PDGF-bb.2: 5’-CGG UCC AGG UGA GAA AGA UUG AGA U-3’,

VEGFc.1: 5’-AUC UAU ACA CAC CUC ACG UGG CAU G-3’,

VEGFc.2: 5’-UAG ACG UUC UCU GCC AGC AAC AUU A-3’,

LIF.1: 5’-GCA ACC UCA UGA ACC AGA UCA AGA A-3’,

LIF.2: 5’-CAC UCU GAC AAA GAA GCC UUC CAA A-3’.

Random Stealth siRNA duplexes coding for non-functional RNAs were used as controls at the same concentrations.

After 4 days of differentiation, osteoclasts were washed 3 times with PBS to remove non-adherent, mononucleated cells. Osteoclasts were then incubated for 20 min at 37°C with 0.25 mM EDTA/PBS and subsequently detached with a cell lifter (Corning Inc., Acton, MA). Osteoclasts were centrifuged at 220g for 5 min at 4°C. Osteoclasts were resuspended in siPORT electroporation buffer (Ambion, Austin, TX). Stealth RNAi duplexes were obtained from Invitrogen (Carlsbad, CA). Predesigned stealth RNAi or scrambled stealth RNAi duplexes (each 800 nM) were electroporated into osteoclasts with a single square wave pulse of 2750 V/cm field strength and 0.4 ms pulse length using a CytoPulse PA-4000 electroporator (CytoPulse Sciences, Columbia, MD). Electroporated cells were resuspended in medium supplemented with RANKL and allowed to recover for 48 h. Osteoclasts and the corresponding conditioned media were then processed for subsequent analysis. Conditioned media were collected, centrifuged and kept at –80°C. The cells were harvested for protein determination and total RNA isolation (Invisorb spin cell RNA mini kit, Invitek, Berlin, Germany) to analyze the knock down efficiencies by Q-PCR.

**RNAi in MC3T3-E1 cells.**

For silencing of murine PDGFR-β and PDGFR-α the following oligonucleotides were used.

PDGFR-β # 1: 5’-UAA GGC UUG CUU CUC GCU ACU UCU G-3’.

PDGFR-β # 2: 5’-UGU AGU UUG AGU CCC UCA UGA UGU C-3’.

PDGFR-α # 1: 5’-UAU GAU GGC AGA GUC AUC CUC UUC C-3’.

PDGFR-α # 2: 5’-AUA AGC UGU ACC UUC GAC CAC UUU C-3’.

Random Stealth siRNA duplexes coding for non-functional RNAs were used as controls at the same concentrations.

Mouse preosteoblastic MC3T3-E1 cells or differentiated osteoblasts, grown at 50-60% confluency, were transfected with 10 nM Stealth siRNA duplexes (Invitrogen Life Technologies. Paisley, UK) using Interferin (Polyplus transfection) reagent. Stealth siRNA were mixed with 125μl transfection reagent. The solution was added to cells. After 48h of incubation at 37°C, the cells were washed, harvested and used in chemotactic assays. A part of the treated cells was processed for total RNA purification (Invisorb spin cell RNA mini kit, Invitek, Berlin, Germany) to analyze knockdown efficiencies by quantitative RT-PCR.

**Quantitative PCR.**

The following primers were used:

Primers (biomers.net)

PDGF-bb Sence 5’-GATCTCTCGGAACCTCATCG-3’

Antisence 5’-GGCTTCTTTCGCACAATCTC-3’

VEGFc Sence 5’-AGCCAACAGGGAATTTGATG-3’

Antisence 5’-CACAGCGGCATACTTCTTCA-3’

LIF Sence 5’ GGCAACCTCATGAACCAGAT-3’

Antisence 5’-ACCATCCGATACAGCTCCAC-3’

CCL9 Sence 5’-CGGGATCCCGCCACCATGAAGCCTTTTCAT-3’

Antisence 5’-CGGAATTCCGTTATTGTTTGTAGGTCCGTG-3’

IL1ra Sence 5’-CCAGCTCATTGCTGGGTACT-3’

Antisence 5’-TTCTCAGAGCGGATGAAGGT-3’

Twgs Sence 5’-TGACGTTCCTGCTGTGTCTC-3’

Antisence 5’-ATTCCGAGGGTTGCACATAC-3’

PDGFR-β Sence 5’-TCAACGACTCACCAGTGCTC-3’

Antisence 5’-TTCAGAGGCAGGTAGGTGCT-3’

PDGFR-α Sence 5’-TGGCATGATGGTCGATTCTA-3’

Antisence 5’-CGCTGAGGTGGTAGAAGGAG-3’

VEGFR-2 Sence 5’-GGCGGTGGTGACAGTATCTT-3’

Antisence 5’-GTCACTGACAGAGGCGATGA-3’

VEGFR-3 Sence 5’-CGAGACTGGAAGGACGTGAC-3’

Antisence 5’-GTACGTGTAGTTGTCCGCCC-3’

LIFR Sence 5’-CCTCATGATCCGACTTCGTT-3’

Antisence 5’-AACCCGGAAAGTGTATGCAG-3’

GAPDH Sence 5’-TCACCACCATGGAGAAGGC-3’

Antisence 5’-GCTAAGCAGTTGGTGGTGCA-3’

RNA amounts were measured with the Ultrospec 3300 pro (Amersham Biosciences, Freiburg, Germany). For each sample, 1μg of total RNA was reversed transcribed into complementary DNA (SuperScript II reverse trascriptase kit) and 1μl of each cDNA was used for quantitative analysis performed in triplicate. Primers pairs to determine gene expression and knockdown efficiencies were purchased from PROLIGO Primers & Probes (France SAS) and from biomers.net (Ulm, Germany). Quantitative RT-PCR were carried out in 25μl volumes using the Multiplex Quantitative PCR System, (MX4000) and the recommended protocol for SYBR Green dye detection method (Brilliant SYBR Green Q-PCR Master Mix, Stratagene, Cedar Creek, USA). The two-step Q-PCRs were set up with a melting temperature of 95°C and annealing-elongation temperature of 55°C for 40 cycles. A dissociation curve program was applied to all samples to check for specific Q-PCR products and avoid primer-dimmer or non-specific Q-PCR products. All transcript levels were normalized to that of GAPDH. Gene expression values were determined by the Ct, defined as the cycle at which the fluorescence of the sample is statistically significant above background.
