# Supplementary figures and images for: Osteoclasts Control Osteoblast Chemotaxis via PDGF-BB/PDGF Receptor Beta Signaling
Source: PLoS One. 2008 Oct 27;3(10):e3537. doi: 10.1371/journal.pone.0003537 (PMC2569415; doi:10.1371/journal.pone.0003537)

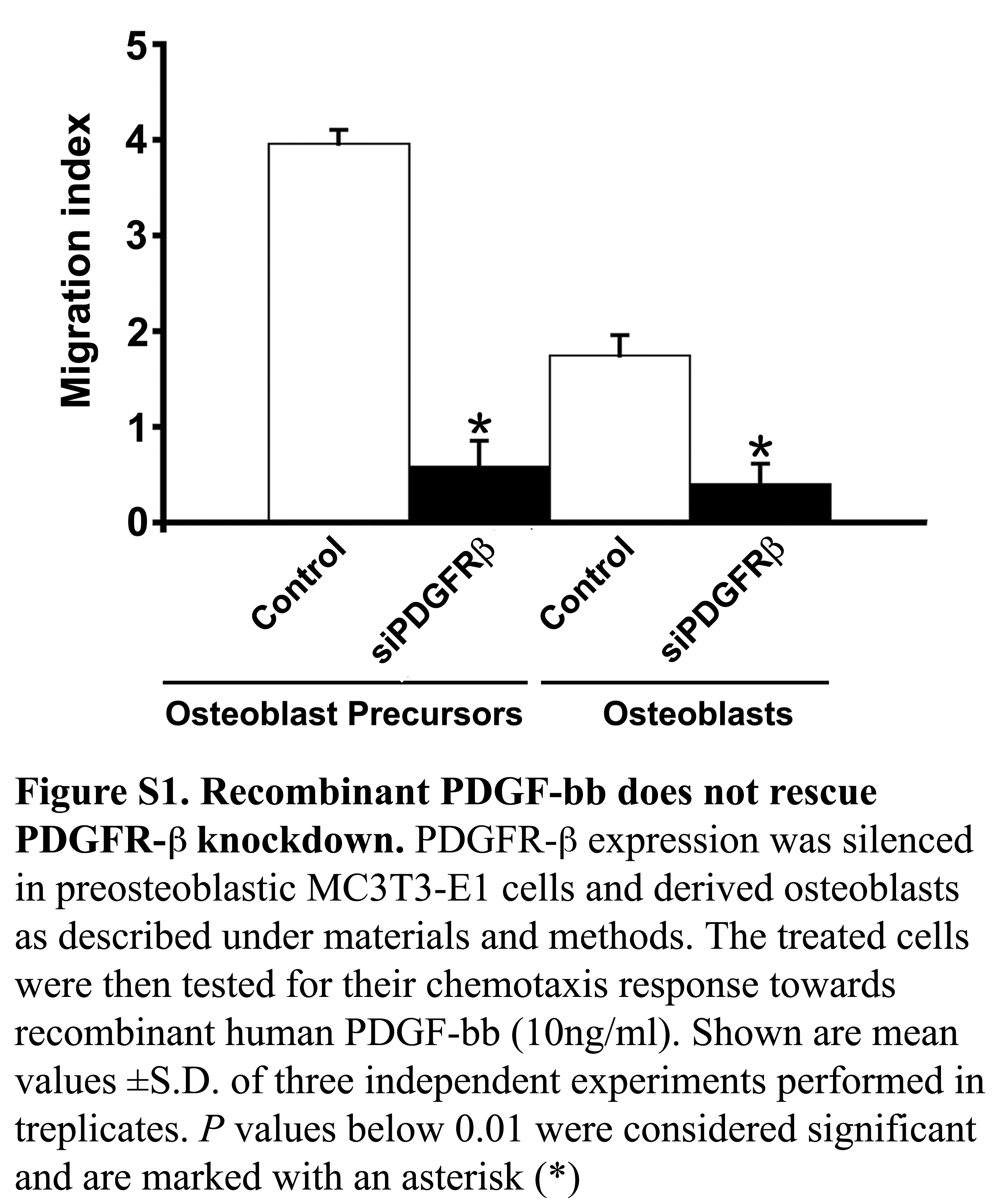

Supplement: Figure S1 — (8.69 MB DOC) [file pone.0003537.s002.doc]
